# The renaissance of acetaldehyde as a psychoactive compound: decades in the making

**DOI:** 10.3389/fnbeh.2014.00249

**Published:** 2014-07-22

**Authors:** Mercè Correa, Elio Acquas, John D. Salamone

**Affiliations:** ^1^Department of Psicobiology, University Jaume ICastelló, Spain; ^2^Department of Life and Environmental Sciences, University of CagliariCagliari, Italy; ^3^Department of Psychology, University of ConnecticutStorrs, CT, USA

**Keywords:** ethanol, catalase, acetate, dopamine, salsolinol, opioids, addiction, drug abuse

As with many events in the history of science, the development of the hypothesis that acetaldehyde is a plausible psychoactive substance with specific central effects (not related to its toxicity) has not been either incremental or progressive. Rather, it has evolved through a process of fits and starts. Initial clinical observations suggesting that accumulation of acetaldehyde could be used as a therapy for alcoholism did not lead to a highly effective treatment, and in fact, it was noted early on that small amounts of ethanol consumed under these conditions (i.e., blockade of aldehyde dehydrogenase) could be perceived as being even more pleasurable (Chevens, 1953). Although some laboratory data in animals appeared at that time (Carpenter and Macleod, 1952), it took a decade for the pre-clinical studies to focus on the potential importance of acetaldehyde. Since Myers proposed in the late 60's that acetaldehyde could be a mediator of some of the effects of ethanol (Myers and Veale, 1969), advances in this field have gone through a push-pull process. His seminal discoveries about acetaldehyde self-administration and neural effects were met with skepticism because of the lack of replicability of results at that time, and the many unsolved questions related to measurements of brain acetaldehyde. In the late 70's, work conducted mainly by Amit's group (e.g., Amit et al., 1977), put acetaldehyde back in the picture. To replicate some of the early discoveries and expand the range of behaviors for which acetaldehyde could be shown to be a mediator of ethanol's effects, this laboratory started to assess the impact of interfering with acetaldehyde metabolism. Again, this work met with considerable skepticism, in part because of issues such as the lack of penetrability of acetaldehyde into the brain due to the high content of aldehyde dehydrogenase (ALDH) in the blood brain barrier, and also the absence of the alcohol dehydrogenase (ADH) isoform that metabolizes ethanol in the brain (Lindros and Hillbom, 1979). More or less at that time, a newly characterized enzyme for the metabolism of ethanol was introduced to the cerebral enzymatic landscape; catalase (Cohen et al., 1980). This enzyme became the focus of the next wave of work by Aragon and Amit related to the acetaldehyde hypothesis (Aragon et al., 1985). *In vitro* and *in vivo* studies, mainly with one pharmacological tool and one genetic mutation, established the first plausible support for the idea that brain ethanol metabolism was possible, and had a clear impact on alcohol consumption (Aragon and Amit, 1993). In addition, late in the 90's the localization of high levels of catalase in catecholamine rich areas in the brain (Zimatkin and Lindros, 1996), identified possible neuroanatomical loci for acetaldehyde formation. Across the first few years of the new century, Aragon and his colleagues developed a series of pharmacological tools that more firmly established the idea that brain catalase activity was directly correlated with ethanol's stimulant effects (Correa et al., 2001). Since then, data have been accumulating at an expanding pace. Additional groups of researchers, most of which are represented in this issue, started to apply different approaches to the “acetaldehyde hypothesis,” which led to a new wave of papers and substantial scientific advances in this field. Genetic, neuropharmacological, electrophysiological and cellular mechanisms for acetaldehyde actions have been established. Potential therapeutic tools have been used. Neural circuits and complex behaviors have been studied, and other mediators of ethanol's actions that depend upon acetaldehyde formation, metabolism, or conjugation have been analyzed.

The present group of articles provides a comprehensive and up-to-date overview of research related to the actions of acetaldehyde, with recent results from the many groups that have decisively contributed to the field. The first review by Peana and Acquas gives a clear perspective on the behavioral evidence showing how acetaldehyde is a neuroactive molecule able to exert key motivational effects that can lead to the development of addiction, and also explains the neurobiochemical correlates of those actions in different intracellular signaling systems and diverse brain areas. A special accent on acetaldehyde formation, metabolism and blockade in relation to its reinforcing characteristics is presented by Diana and colleagues. Emphasis is put on the use of thiol compounds, which sequester acetaldehyde, as therapeutic agents preventing those effects. A very interesting methodological approach using gene-specific manipulations to change enzymatic activity, both in the liver and in the brain, has led Israel and colleagues to explore the behavioral consequences of central versus peripheral acetaldehyde metabolism. Direct administration of ethanol or acetaldehyde either in the periphery or in the brain, as employed in the studies from Correa and Salamone's lab, describe c-Fos expression (a marker of neural activity) across a broad range of brain areas involved in the motivational actions of both compounds. At the molecular level, Weiger and colleagues present data indicating that acetaldehyde acts on maxi calcium-activated potassium channels, a key factor in electrical stimulation of neural cells.

The role of acetaldehyde/monoamine conjugates that form tetrahydroisoquinoline alkaloids is presented in the next group of articles. Special attention is paid to the conjugate of acetaldehyde with dopamine, salsolinol. This molecule has demonstrated to be central for the actions of ethanol in the mesolimbic system (Melis et al., 2013). Electrophysiological data presented by Xie, Ye and colleagues show how salsolinol modulates firing of dopamine neurons in brain slices from posterior ventral tegmental area via activation of opioid receptors located in neighboring GABAergic neurons. These data are discussed by Polache and Granero in a commentary, written from the perspective of their own extensive research on salsonilol-induced dopamine release in the accumbens in behaving animals. Deehan and Rodd, report on the role of salsonilol and other conjugates in acetaldehyde self-administration, in an integrative review of human findings and their own key animal data involving the mesolimbic dopaminergic system.

Although dopamine has been a major focus for studies of acetaldehyde's central actions, other neural systems are also involved. For instance, the role of the opioid system in the neurochemical and behavioral effects of acetaldehyde is reviewed by Font and Pastor, who not only discuss the effects of salsonilol on *mu* opiod receptors, but also summarize what is known about acetaldehyde formation in brain nuclei rich in catalase and beta-endorphine neurons. Elegant studies conducted by Cannizzaro's laboratory demonstrate that the endocannabinoid system modulates not only operant acetaldehyde self-administration, extinction, and relapse, but also its effects on the emission of punished responses during conflict experiments, all of which are key features of addiction. A relevant role for acetate, a metabolite of acetaldehyde, as a mediator of the sedative and ataxic effects of alcohol is presented by Correa and colleagues. These results are hypothesized to be mediated by the neuromodulator adenosine.

The work by March, Molina and colleagues is focused on a particulary important neurodevelopemental period; early ontogeny. At this stage, drugs have a substantial impact on the nervous system, and in the case of ethanol, its actions can be affected by the fact that rats show a high rate of brain catalase activity specifically in this period. Thus, these authors provide extensive results about vulnerability to abuse and addiction in newborn and infant rats, which is induced by early ethanol and acetaldehyde exposure. These data are discussed in a commentary by Zimatkin, an expert on brain enzymatic localization, in terms of the ideal scenario in brain pups for acetaldehyde accumulation (high catalase-low ALDH). Finally, on the other extreme of the neurodevelopmental process, Vaglini, Corsini and colleagues review data on the impact of acetaldehyde on Parkinson's desease. In this case the authors focus on the role of the other important brain enzymatic system for the central formation and degradation of acetaldehyde; cytochrome P450-2E1, which is highly concentrated in substantia nigra pars compacta, an area rich in dopamine cell bodies. Acetaldehyde acting as a substrate/inhibitor of this enzyme, may facilitate the susceptibility of these dopaminergic neurons to toxin-induced parkinsonism.

Many more exciting discoveries are yet to come, and new methodologies still need to be developed in order to overcome the obstacles that continue to obscure our understanding of the role of acetaldehyde. Especially important would be the need for reliable measurements of central acetaldehyde production under physiological conditions. However, we can certainly say that acetaldehyde, its metabolites and its conjugates are critical molecules for the complex set of effects observed after ethanol consumption, especially those related to the development of its pathological consumption.

## Conflict of interest statement

The authors declare that the research was conducted in the absence of any commercial or financial relationships that could be construed as a potential conflict of interest.
